# Predictors of COVID-19 Mortality in Residents of Flint, Michigan: Effect of Age, Gender, Smoking, and Health Plan

**DOI:** 10.1017/ash.2021.111

**Published:** 2021-07-29

**Authors:** Mariam Younas, Danielle Osterholzer, Philip McDonald, Carlos Rios-Bedoya, Ghassan Bachuwa, Sherry Demian, Smit Deliwala, Lalida Kunaprayoon, Harini Lakshman, Thulasi Beere, Uyoyo Omaduvie

## Abstract

**Background:** Current literature suggests that older age, hypertension, and diabetes mellitus confer a significant increased risk of mortality among patients with COVID-19. The purpose of this study is to further characterize the predictors of mortality in patients with COVID-19 in residents of Flint, Michigan, based on variables such as gender, age, smoking status, health insurance plan, and comorbidities. **Methods:** Hurley Medical Center, is a 443-bed public, nonprofit, teaching medical center located in Flint, Michigan. In total, 289 consecutive adult patients (aged ≥18 years) with confirmed SARS-CoV-2 infection by nasal polymerase chain reaction (PCR), admitted and discharged from our facility from March 2020 through June 2020, were retrospectively analyzed. **Results:** During the 4-month study period, the overall in-hospital case fatality rate (CFR) was 18% (51 of 289), with highest CFR in the age group aged 60–69 years (36%; *P = .*06). Nonsurvivors tended to be older with mean age of 67 years (95% CI, 61.6–71.8) versus survivors with mean age of 60 years (95% CI, 57.7–62.4). Highest mortality was seen in patients with Medicare or Medicaid as their sole health plan (39%, *P =* .59). Men comprised 51% (148 of 289) of the cohort with CFR of 21% versus 14% in females. Females tended to be younger with a higher body mass index (BMI) than their male counterparts (mean age of 58 years, mean BMI of 35 in women vs a mean age of 62 and BMI of 29 in men). A higher proportion of deceased were active smokers (51%; *P* = .02). CFR was highest in patients with hypertension (92%,; *P* = .15), followed by diabetes (44%; *P* = .85), chronic kidney disease (CKD) (31%; *P* = .10), obstructive sleep apnea (OSA) (28%; *P* = .25), asthma (22%; *P* = .64), and coronary artery disease (22%; *P* = .34). It was lowest in patients with end-stage renal disease (3%; *P* = .69). **Conclusions:** Our study suggests trends towards higher mortality with male sex, hypertension and diabetes, along with other comorbidities. Smoking seems to be a strong predictor of mortality in this cohort. Further studies are needed to ascertain the relationship between possible risk factors with COVID-19 mortality in residents of Flint, Michigan. Describing and understanding the potential risk factors is the key to improving outcomes in this population.

**Funding:** No

**Disclosures:** None

